# Making it “work”: mothers’ perceptions of workplace breastfeeding and pumping at Dutch universities

**DOI:** 10.1186/s13006-021-00433-w

**Published:** 2021-11-08

**Authors:** Maike Hentges, Eva Pilot

**Affiliations:** Department of Health, Ethics and Society, Care and Public Health Research Institute (CAPHRI), Faculty of Health, Medicine and Life Sciences (FHML), Maastricht University, Maastricht, The Netherlands

**Keywords:** Breastfeeding, Pumping, Mothers, Workplace, Universities, The Netherlands

## Abstract

**Background:**

Dutch breastfeeding rates are below World Health Organization’s recommendations and targets despite the benefits for individuals and society. Increasing the rates is complex due to multiple breastfeeding determinants, of which maternal education and employment are dominant. This study aimed to identify the perceptions and experiences of mothers employed at Dutch universities regarding barriers and enablers to workplace breastfeeding and pumping.

**Methods:**

The study adopted a descriptive, qualitative research design. Thirteen semi-structured online interviews, underpinned by the Social Ecological Model, were conducted in 2020 with three experts and ten academic employees from five universities who had breastfed or pumped at work within the past five years. Qualitative data were examined through a thematic analysis.

**Results:**

Four main themes were identified: physical work environment, social support, work culture and organisation, policies and legal rights. Most mothers had more negative than positive experiences combining breastfeeding with work. They were unable to exercise their rights as a breastfeeding employee due to inappropriate and inaccessible lactation rooms, a lack of communication and information-provision, other people’s lack of awareness, inflexible working hours and unadjusted workloads, especially for teaching positions. All participants found the duration of Dutch maternity leave too short.

**Conclusions:**

Universities need to increase institutional efforts at multiple levels and meet their legal obligations to support breastfeeding employees. Workplace interventions should be combined with more political commitment to normalise breastfeeding, monitor compliance with maternity protection provisions at work and prolong parental leave to encourage breastfeeding continuation.

## Background

Despite the benefits for mothers and infants, breastfeeding duration in the Netherlands is below World Health Organization’s (WHO) recommendations stating that six months of exclusive breastfeeding, followed by complementary feeding until two years or longer, are necessary for optimal development [1, 2]. Previous research determined that breastfeeding protects the newborn against infectious and noncommunicable diseases, while reducing the mother’s risk for chronic conditions [3–6]. Increased breastfeeding rates result in substantial savings for healthcare systems, making breastfeeding promotion an essential public health intervention [7, 8]. Therefore, the WHO Health Assembly set the target to increase the global six months exclusive breastfeeding rate to at least 50% by 2025 [9].

Although Dutch six months exclusive breastfeeding rates have more than doubled from 18 to 39% between 2010 and 2015, numbers remain below the WHO’s targeted minimum, indicating that the Netherlands needs to enhance breastfeeding promotion to reach this target within the next four years [2]. While the majority of women in the Netherlands intended to breastfeed exclusively at childbirth, only 64% of infants were exclusively breastfed one week later. This percentage decreased to 47% three months postpartum, resulting in a median exclusive breastfeeding duration of eight weeks. The fact that 70% of mothers mentioned health benefits as the primary reason to breastfeed, over 20% more compared to 2007, implies greater knowledge, but also that women face barriers to meet their intentions [2].

The two-fold increase in Dutch exclusive breastfeeding rates and the heightened awareness might be attributable to (inter)national initiatives. Although certification has stopped in the Netherlands [10], the “Baby-Friendly Hospital” certificates were accredited to 93% of Dutch hospital and maternity facilities promoting exclusive breastfeeding [11]. The Netherlands further ratified the “International Code of Marketing of Breast-milk Substitutes”, but has implemented few legal provisions and monitoring mechanisms [12, 13]. Despite no national plan for breastfeeding promotion [11], the Netherlands Nutrition Centre and the National Breastfeeding Council undertake initiatives, including the recent “Breastfeeding works!” (*Borstvoeding werkt!*) campaign informing employers about breastfeeding-friendly work environments [14]. Nevertheless, the inability to reach satisfactory breastfeeding rates is of concern and suggests that challenges for effective interventions exist.

To analyse this issue, numerous studies investigated the factors determining mothers’ breastfeeding decisions and found that especially mothers’ education and employment could influence breastfeeding initiation and duration [2, 15–18]. Accordingly, maternal tertiary education resulted in significantly higher chances of continued breastfeeding at hospital discharge [17] and was associated with a decreased likelihood of early complementary feeding [15]. In the Netherlands, more educated women initiated breastfeeding more frequently (90%) than less educated (69%) and continued longer [2, 18]. However, the WHO Regional Office for Europe [1] stated that “policies in the workplace and the employment market” contribute to breastfeeding discontinuation, making exclusive breastfeeding difficult for mothers who return to work early, regardless of their socio-economic status. Comparing maternity protection provisions between EU-27 countries demonstrates that paid Dutch maternity leave of 16 weeks (10 weeks postpartum) is relatively short [19, 20]. Dutch research suggests that struggles with combining breastfeeding and employment were a major reason for ceasing exclusive breastfeeding [2], and the odds of breastfeeding continuation after four months were 57% higher for Dutch women working a maximum of 16 h per week [18].

Although the *Voedingsrecht* of the Dutch Working Hours Act [21] obliges employers to provide appropriate lactation spaces and enable women to use 25% of their paid working time for breastfeeding in the first nine months, a lack of workplace support to comply with these rights was identified [22]. Since mothers in the Netherlands are increasingly working the same hours as before childbirth and more than half work at least 28 h per week [23], more workplace support to encourage breastfeeding must be implemented, but there remains a paucity of evidence on Dutch workplace breastfeeding. Therefore, this study aimed to describe mothers’ perceptions regarding enablers and barriers to breastfeeding and pumping at work. Considering the lack of Dutch research in this target group and the discrepancy between highly educated mothers’ intentions and breastfeeding duration, academic mothers employed at Dutch universities were targeted. Thus, the research set out to answer the question of how mothers employed at Dutch universities experience workplace breastfeeding and pumping. Additionally, experts in the topic of breastfeeding were consulted and recommendations for employers, employees and policy-makers were developed to inform breastfeeding promotion.

## Methods

**Fig. 1:**
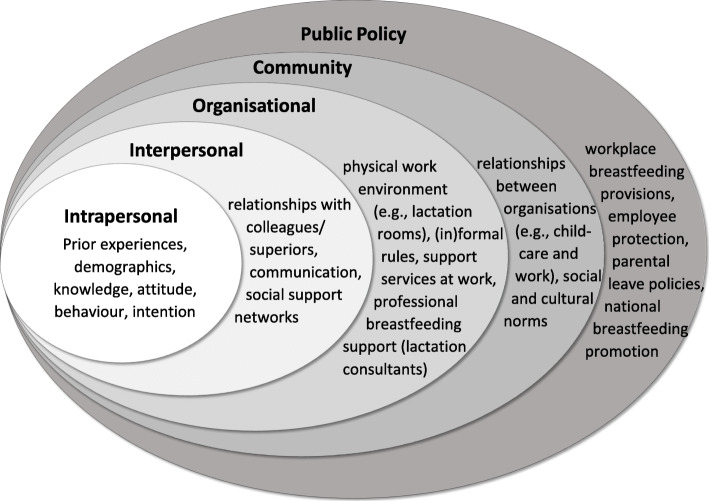
Exemplification of applying the Social Ecological Model to workplace breastfeeding and pumping. Source: Created by authors based on Bronfenbrenner [25]

A descriptive, qualitative research design incorporating a phenomenological inquiry from a social constructivist perspective was chosen, thereby assuming that women’s lived experiences are subjective and context-specific [24]. To capture complex breastfeeding determinants, this study was underpinned by the Social Ecological Model which argues that five interrelated dimensions (intrapersonal, interpersonal, organisational, community, public policy) influence health behaviours [25, 26]. Figure 1 Exemplifies how these dimensions can relate to workplace breastfeeding and pumping.

### Participants

The target population was female academic staff at 14 Dutch research universities being part of the Association of Universities in the Netherlands [27] and breastfeeding experts (i.e., lactation consultants, researchers). Academic staff included associate/assistant/full professors, lecturers, PhD candidates and research staff, of which the latter two are mostly on time-bound contracts and the biggest groups at Dutch universities [27, 28]. The share of female employees is considerably larger for research staff (46%), PhD candidates (43%) and lecturers (41%) compared to senior lecturers (29%) and full professors (20%) [29].

Eligible participants were healthy mothers of healthy children to exclude contraindications to breastfeeding. Since the literature points towards a cultural effect on breastfeeding, native Dutch and non-Dutch women were included [15, 30]. Eligible women were not on maternity leave and were currently breastfeeding or had breastfed or pumped at work within the past five years. Given the proposed effect of working hours, both full-time and part-time employees were included [18]. All participants needed a good command of English.

Participants were recruited through purposive and snowball sampling [24]. Websites of Dutch breastfeeding consultant associations and the *Kenniscentrum Borstvoeding* were searched for lactation consultants and researchers, while 12 heads of department at six universities were initially contacted to recruit academic staff. Contacts were called or approached via email with an attached information letter and research announcement. Sampling was ongoing while data collection and analysis was undertaken concurrently until data saturation had been reached [24, 31]. The final sample consisted of ten academic employees (mothers) and three experts (two lactation consultants, one author on workplace breastfeeding in the Netherlands).

### Data collection

From the end of March until May 2020, data were collected through single, semi-structured online interviews conducted in English. Participants were informed about the study, their rights and personal data use prior to interviews. Before verbally consenting to participate and be recorded, remaining questions were clarified by the researcher. On average, expert interviews lasted 64 min and employee interviews lasted 44 min.

Open-ended questions for mothers were structured according to the Social Ecological Model ranging from the participant and her family to the social, organisational and physical work environment, and policies protecting breastfeeding employees (e.g., *How would you describe the influence of your social workplace environment on your breastfeeding experience?*). Expert interviews focused on challenges of workplace breastfeeding, interventions and policies (e.g., *What is your opinion about the Voedingsrecht and how do you perceive it to work in practice?*). The interview guides were supplemented as additional files (see Additional file 1). Following a separate test study with a lactation consultant prior to data collection, questions about hygiene, national breastfeeding recommendations and mental health were added. A reciprocal interaction and member-checking through probing questions improved the interviews [24, 32].

Recorded interviews were transcribed verbatim. Transcripts were stored on an encrypted hard drive and sent to participants for verification, after which recordings were deleted. Confidentiality was safeguarded through anonymising identifiable information of participants (i.e., assigning numbers plus “A” for academic employee and “E” for expert) and their workplace.

### Data analysis

Data from experts and mothers were pooled and analysed together. Interviews were manually coded following Braun and Clarke’s [33] six non-linear phases of a thematic analysis, starting with data familiarisation and open coding of aspects relevant to the research question. Codes were compared and clustered into colour-coded (sub-)themes, generating a thematic map. Themes were reviewed against the associated codes and the whole data set to identify suitability and potential overlaps. Codes and themes were clearly defined, followed by a final documentation of results with selected quotations from participants. To increase credibility, disconfirming responses were acknowledged and a codebook was established [24, 31]. Data saturation was reached as no new codes were developed when analysing the 12th interview. A final interview was performed to validate saturation. Participants received the opportunity to review the findings and the interpretation.

## Results

Ten employees with diverse disciplinary backgrounds from five universities participated. Table 1 depicts employees’ sociodemographic characteristics, breastfeeding and the timing of postpartum return to on-campus work. On average, mothers were 34 years old and returned to on-campus work after 16 weeks, the longest period being eight months for one mother working from home. Two identified with a non-Dutch nationality, more than half worked 32 h per week and had full-time employed partners. Women who extended their leave beyond the statutory 10 weeks postpartum breastfed longer, but no difference was observed between working hours. Four themes with several sub-themes were identified as illustrated in Table 2 : (1) physical work environment, (2) social support, (3) work culture and organisation, (4) policies and legal rights. These (sub-)themes were applied to structure the results.

**Table 1:** Characteristics of participants

Participant ID	Age	Nationality	Education	Occupation	Working hours/week (permanent/ fixed-term contract)	Marital status (full-time/part-time working partner)	Age child (ren)_**b**_	Any BF duration in months (of which exclusive)_**c**_	On-campus return in weeks_**c**_
**1E**		Dutch	–	Author, pre/postnatal yoga teacher	–	–	–	–	–
**2A**	32	Dutch	Postgrad.	Teaching fellow, PhD student	20_a_ (n/a)	Domestic partner (full-time)	1	11 (4)	9
**3E**		Dutch	–	Lactation consultant	–	–	–	–	–
**4E**		Dutch	–	Lactation consultant	–	–	–	–	–
**5A**	36	Greek	PhD	Assistant professor	40 (fixed-term)	Married (full-time)	2	18 (6)	22
**6A**	38	Dutch	PhD	Postdoc. researcher	16 (fixed-term)	Married (part-time)	7 5 2	- n/a 23 (4)	32
**7A**	41	Dutch	PhD	Assistant professor	32 (permanent)	Married (full-time)	18 months	14 (4)	20
**8A**	35	Dutch	PhD	Assistant professor	32 (permanent)	Domestic partner (part-time)	9 5 1	- 7 (n/a) still (5)	n/a 13
**9A**	41	Dutch	PhD	Assistant professor	32 (permanent)	Single	4	still (6)	16
**10A**	30	Dutch	PhD	Postdoc. researcher	32 (fixed-term)	Married (full-time)	7 months	still (6)	16
**11A**	32	Dutch	Postgrad.	Teaching fellow	24 (permanent)	Married (full-time)	4 2 8 months	12 (6) 9 (5) still (6)	12 10 20
**12A**	30	Dutch	Postgrad.	Junior researcher, PhD student	32 (fixed-term)	Married (full-time)	5 months	still exclusive	10
**13A**	29	Nepalese	Postgrad.	PhD student	32 (fixed-term)	Married (full-time)	6 months	still (5)	12

*Note.* n/a = no information provided; BF = breastfeeding; A = academic employee. Participant IDs were assigned according to the interviews’ chronological order

_a_ 40–50 h/week in practice as PhD is not contracted

_b_ In years if not indicated otherwise

_c_ For those children aged ≤5 years where mother breastfed/pumped at Dutch university

**Table 2:** Summary of main themes and sub-themes

Main theme	Sub-theme
Physical work environment	Availability and accessibility of lactation rooms Lactation room quality Pumping in office rooms Cleaning pumping equipment and storing breast milk
Social support	Social perceptions of breastfeeding Characteristics of social work environments Communication Childcare
Work culture and organisation	Flexibility of working hours Workload and output expectations Being a mother and an employee
Policies and legal rights	Knowledge of policies and legal rights Exercising rights Maternity leave

### Physical work environment

#### Availability and accessibility of lactation rooms

Lactation facilities at work impacted mothers’ experiences and their ability to continue breastfeeding. Although universities had designated lactation rooms, mothers reported problems with their availability and accessibility. Employees had to book time slots in advance, which was difficult when meetings unexpectedly lasted longer and there were other breastfeeding employees with similar pumping routines. Four women from two universities stated there was only one room at their faculty:

*“We have to share the room with all the other mothers*. *.. but also it’s a meditational, relaxational room, so the other users want to use it as well…. it’s really busy at the same time, because the mothers want to use it in the morning and during lunch and at the end of the afternoon. And when your meeting is overtime and then- then you really come into trouble with the- with the schedule of the pumping room. So that’s really difficult.”* (10A).

Consequently, designated rooms were booked, multiple women pumped together or mothers had to wait for the previous person, who was then pressured to finish. This was perceived as stressful and frustrating, which could decrease milk production. After raising the issue with Human Resources, one participant reported they provided access to a basement storage room and suggested room dividers for simultaneous pumping. In contrast, one faculty had two lactation rooms next to each other but booking ahead was still necessary.

Further, mothers needed to get the key from, and bring it back to, secretaries, which contributed to time issues if rooms were distant and hard to reach. Therefore, meeting or storage rooms were occasionally used, although these were unsuitable and unpleasant due to lack of facilities, cold climate, or privacy issues. Having to rely on secretaries for access was perceived as discouraging, making one mother feel “*watched*”. Describing the accessibility as a “*nuisance*”, one participant stopped pumping at work:

*“It was a bit frustrating always to find a room, you know, you always had to go to talk to the- to the receptionist. .. It would have been nice that you know, ok, I can go there, I can close it, I can relax. .. I really was fed up with all the hassle and I decided, ok, I just quit, I’ll stop, I don’t want it anymore.”* (7A).

#### Lactation room quality

Given distant locations, some mothers stored their bottles in the department’s fridge, reduced their lactation breaks, and many chose to or had no other option than pumping in their office, which related to poor quality of lactation rooms. Experts summarised that the *Kolfcode* obliges employers to provide a lockable room with good climate and comfortable seating. Electricity, a sink, fridge and cupboard were favourable, but rarely supplied. Reflecting experts’ hygiene concerns, three women reported no nearby access to warm running water and others found the spaces unclean:

*“It wasn’t possible to clean. .. the table that I was using.. .. I then took a little bottle of water that I could use to clean the table, which is also not the most hygiene. .. the stains that were on the table that I made, they’ve been there all the time, so I never saw*- *saw some cleaning activity signs there.”* (7A).

Half of the participants utilising lactation rooms were dissatisfied with the interior and some designated spaces were storage rooms or classrooms with old office furniture. Even if rooms complied with the *Kolfcode*, relaxing environments to stimulate the let-down reflex through soft lightning, warm climate, comfortable seating and shielded spots were hardly facilitated:

*“It* [room] *just looks horrible. Like it’s an old hospital bed, it’s got neon lighting, it’s very cramped, there was a chair there that had some white stains on it, probably milk from some mothers.. .. it’s quite important to be relaxed, and so it was really not a relaxing environment at all.”* (2A).

Two mothers suggested homelike atmospheres through couches, plants, music or reading material. Others stressed that pumping was energy-consuming, emphasising the importance of a bed, although time pressures would not allow to rest. Those struggling with the milk flow due to inappropriate environments looked at photos or videos of their babies as advised by lactation specialists or colleagues. In contrast, two mothers claimed that they were able to pump in almost any space and some remarked that rooms did not need to be “*special*”, although all agreed they must be safe, private, clean and easily accessible. Three participants were satisfied with the provided rooms as they were furnished appropriately.

#### Pumping in office rooms

Six employees pumped in their office due to convenience or no better room being available. This was positive for mothers sharing their office with supportive, female colleagues, but some indicated obstacles such as large windows. Where doors were not lockable, two attached a “*Do not disturb*” note. One mother preferred lactation rooms to physically separate pumping from work, as pumping was a relaxing moment to feel connected with her child:

*“Your baby is a lot on your mind. And sometimes it feels very stressed. .. And within the meantime your colleagues are talking and you’re still working and there’s a lot in your head. And it helped me also to- to have a set time to think about the baby. .. afterwards I could let go and then focus on my work. .. it was relaxed to have a moment on your own.”* (12A).

#### Cleaning pumping equipment and storing breast milk

Many women had to use common department facilities for cleaning pumps and storing breast milk. Two respondents pointed out that shared fridges were often full and many found them unclean. Mothers were worried about rinsing their equipment in the kitchen, not only due to hygiene concerns, but also because it exposed something personal:

*“Everyone can kind of access it and it feels very private. Like it’s your own body fluid, and it’s the feeding for your baby. And the idea that someone touches it, it’s already kind of weird.”* (6A).

One participant felt especially uneasy doing it around male colleagues and respondents did not want to embarrass others, which was associated with (perceived) social norms regarding breast milk:

*“I can imagine that people don’t think it’s hygienic to rinse your stuff.. .. it might be the same as rinsing blood in your - in your kitchen that you’re sharing, right?*. .. *even though, of course, that’s- that’s clean stuff.”* (7A).

To prevent discomfort and for hygiene reasons, four women hid bottles in bags. One participant attached a “*Don’t touch it*” note, while another used her own cooler bags. Some avoided cleaning in the kitchen by briefly rinsing the pumps, going to bathrooms, or using cloths to absorb the milk. In contrast, two mothers did not consider it necessary to hide their milk, and one respondent regarded it relevant to normalise breastfeeding by making it visible:

*“I did wash my, all this- this pumping stuff in the pantry. But because I also felt it’s important to show that you pump. .. you hardly ever see that. And then for new, young mothers. .. I thought early on that it’s important to show that you’re breastfeeding.”* (9A).

### Social support

#### Social perceptions of breastfeeding

Although some women emphasised how easy breastfeeding and pumping was, others reported problems, including pain, pressure on breasts, or not expressing as much milk as through breastfeeding, particularly under time pressures. Half indicated that their baby sometimes refused the bottle, which could be stressful when returning to work. Two stated that breastfeeding initially took eight hours daily, making it an “*extra job*” that was no automatic process:

*“We have the idea that it’s very natural and very simple, that it’s easy to do.. .. it’s hard in the beginning, it’s really a skill that you have to learn.. .. it takes so much energy. .. you’re producing the food of an entire, quickly growing human being.”* (1E).

However, interviews suggested that women’s social environment, particularly men, might not be aware of how physically and mentally intense it can be. Some experienced that (public) breastfeeding was not normalised, making one expert claim that the Dutch society was not “*pro-breastfeeding*”. One participant reflected on breastfeeding at the end of her conference presentation, making her feel as if she was doing something wrong because of explicitly positive responses:

*“The way they kind of responded to it, like by ensuring that they were ok with it, kind of made me feel weird.* *People were like, ‘Ah, you’re such a role model.’ But it made me feel very uncomfortable, ‘cause it made me feel like it was not normal.* *They are giving me permission for something I thought I didn’t need permission.”* (6A).

Similarly, one colleague who breastfed at work while marking papers was called a “*supermum*”. Thus, rather than being described as a role model, support and acknowledgement should come through making breastfeeding part of the (working) culture:

*“It should not be a social dogma or social problem saying that breastfeeding is something not as a work part. It’s a part of daily life and it should be taken along accordingly.”* (13A).

#### Characteristics of social work environments

Mothers’ experiences differed with the sex and age of colleagues and superiors. While most employees in two departments were male, four stated the majority was female, including young mothers. Although participants made it explicit that others accepted workplace breastfeeding, those in male-dominated environments perceived less encouragement, thus feeling more uncomfortable and stressed. One employee claimed that she “*didn’t experience any support*” and felt left alone. Those mothers feeling disconnected to others did not discuss the problems they encountered:

*“My head of department was an older male that I felt a bit distanced from so maybe that’s also not someone that I would ask about breastfeeding. I think if my head of department would have been someone that I related more or someone that I felt more comfortable with, I might have discussed this.”* (2A).

One participant elaborated that her decision not to talk about breastfeeding with men could be her “*fault*” given her cultural background. Experts believed that women in male-dominated environments were more inclined to stop pumping at work, although two employees perceived understanding where the head of department was a young father. Mothers in female-dominated departments where breastfeeding was normalised felt “*lucky*” and empowered:

*“They really accept it. The supervisor is,. .. and the whole department as well, very emancipated. So, there I’m feeling really. .. stimulated for breastfeeding.”* (11A).

However, one expert remarked that older female superiors who had breastfed could set standards that they expected to work for others, thus overlooking individual needs.

#### Communication

Respondents were more comfortable discussing their plans or problems with people towards whom they felt sympathetic, which was related to women feeling “*vulnerable*” after pregnancy. Experts proposed that mothers might additionally be concerned about job security. One mother suggested that primiparous mothers felt specifically insecure about negotiating with superiors and many participants did not want to “*complain*” about experienced barriers, particularly when no other breastfeeding employees expressed problems:

*“Everyone kind of said, ‘Ok, no, this is how it is and we accepted it’, which maybe also led to me accepting it instead of going to HR* [Human Resources] *and saying like, ‘Ok, this is my right,. .. why can I not make use of this right?’. .. You don’t want to be the only person complaining if others aren’t.”* (2A).

One mother explained that some might not see themselves in the position to complain, reflected by another participant who felt like the “*black sheep of the department*” after expressing her struggles with Human Resources. Some highlighted that even if they wanted to discuss any issues, they did not know whom to approach.

Experts advised mothers and employers to be proactive in discussing plans to diminish stress, but only three participants talked about breastfeeding with superiors or Human Resources prior or upon return. While some indicated superiors’ and colleagues’ lack of awareness about their breastfeeding, one interviewee perceived it unnecessary to communicate about it because workplace breastfeeding was normalised. Interviewees missed that nobody checked on them after returning, making them feel as if universities had no interest in their well-being and did not consider it valuable that employees continued breastfeeding. Some mothers reflected on requesting to leave meetings earlier for pumping and emphasised the necessity of providing an environment where employees could raise their need to breastfeed without feeling that it was inappropriate:

*“Sometimes I have to say that, well, I have to leave the meeting at this time slot because I have to pump. And he’s* [superior], *he’s. .. not that supportive.. .. He makes kind of strange comments, ‘Well, I can’t help you with that’. .. but he accepts it. .. but yeah. Not encouraged.”* (10A).

Concerning the communication with and support by colleagues, the majority was positive and underlined the importance of sharing advice and frustration. Three women discussed issues in online chat groups with mothers in their departments. Nevertheless, it was remarked that it should not be colleagues’ responsibility to provide extra support if not facilitated institutionally. Without employers’ endorsement, mothers perceived it was their responsibility to solve problems. However, referring to their legal right, many believed that workplace breastfeeding was an institutional issue requiring collective action.

#### Childcare

At the community level, interviewees debated the organisation of childcare. One university offered on-campus day-care, enabling the participant to breastfeed directly, but others organised childcare externally. When discussing whether on-campus childcare might have impacted their experience, opinions differed. Some mothers and all experts regarded on-campus childcare beneficial for breastfeeding, particularly for bottle refusers, although having the child nearby could distract from work:

*“. .. if it* [day-care] *would be at my workplace it would be easier to feed her, but also it would give more distraction, I think.”* (12A).

Yet, all suggested that going to the child to feed, occasionally bringing it to the office or working from home made infant feeding easier.

### Work culture and organisation

#### Flexibility of working hours

The majority of women perceived the organisation and nature of their work as a main barrier to continued breastfeeding. Although a few remarked that academic work was comparatively flexible, many reported difficulties in creating time for breastfeeding, especially where fixed schedules overlapped with lactation breaks. To keep up the milk production, regular pumping matched with the child’s feeding was important but unrealisable. Women spent approximately 30 min per lactation break, two or three times daily, but some reduced the frequency, postponed and skipped sessions, or ceased pumping due to time issues, which could impede breastfeeding at home. Consequently, one mother recalled breast pain during overtime meetings, one child did not drink sufficiently following a changed feeding routine, and two introduced formula. Teaching positions were less flexible than research positions, making it impossible to use the 25% working time:

*“It became really difficult. Because I had a block of two hours. .. after each other. .. then you have these 15 minutes breaks and then you have to make them larger. .. I don’t wanna tell my students that I’m pumping milk, that’s weird.. .. the whole* [pumping] *schedule became off.”* (8A).

Consequently, some employees pumped in classrooms in between, built up a milk supply at home, read papers, made phone calls and responded to emails while pumping. One participant negotiated to reschedule her teaching with her superior who advised to allocate one day per week solely for teaching. One expert commented on superiors who suggested compensating the missed 25% by taking days off:

*“That’s like saying to someone I need to pee and then you say, ‘Wait until the weekend and then you can take the whooole weekend to pee.’ You know, it’s not how it works!”* (1E).

#### Workload and output expectations

Despite reduced contracts after maternity leave and requiring additional time for breastfeeding, workload and output expectations mostly remained the same, contributing to overtime and stress, sometimes at the expense of women’s sleep and milk production. Two noted that schedules were not recalculated to account for 25% breastfeeding time, exacerbating the situation on teaching days. Teaching hours of one mother even increased, a significant obstacle for which she received little understanding. To compensate for a missed teaching peak during maternity leave, one mother returned to work earlier and worked more, making her cease pumping to save time:

*“You still have to do the- the same amount of work. .. if that would be adjusted, then I think that would make it much easier for, yeah, for mothers who do want to breastfeed. To also continue to do that.”* (2A).

Instead, two respondents negotiated a gradual return with fewer responsibilities and colleagues taking over workloads, although others argued that sharing workloads was difficult. This contributed to work accumulating during maternity leave and additional pressure. Nevertheless, one mother was positive about the university’s working culture where everyone was individually responsible, which also allowed two women to work from home. Further, an interviewee underlined that probably nobody expected extensive publications immediately, and that it was in the nature of an academic career to fulfil expectations rather than insisting on legal rights. However, academia’s focus on outputs also led to overtime:

*“I still work at my free days.. .. it’s really bad that they don’t explicitly mention that.. .. Well, at least they can say something about it.. .. that would be helpful, just to acknowledge that. .. But I think it’s-- that’s not how the scientific and academic world works. .. they are also looking at your output and if you don’t have a paper each year, they notice.”* (10A).

#### Being a mother and an employee

Due to these obstacles, mothers experienced the transition as “*difficult*” or “*horrible*”, making them feel “*guilty*” and insecure, although a few enjoyed being back. Some perceived pressure to function as before:

*“During pregnancy, you know, you have to prove that you are not sick, and when you’re a mother you have to work like you don’t have any kids.”* (1E).

This pressure was external given demanding work, but also internal. One employee had self-doubts given conflicting discourses around highly educated mothers who focus on childcare:

*“The kind of rhetoric like a woman should be kind of earth mother, being home with their child, and. .. the critic that you have this highly educated woman who is then only a child-bearing woman, so it’s constant all these values.”* (6A).

Some women tried to separate being a mother from being an employee, but workplace breastfeeding combined both and interrupted the workflow. Half indicated it was impossible to completely be at work with their child on their mind, making roles inseparable. The combination became challenging for those (planning on) going to conferences off-campus. While two employers covered travel costs for family members to support breastfeeding, one mother experienced no understanding when requesting to bring her child. Another mother added that paying other’s tickets did not turn into institutional policy out of financial reasons, which was “*structurally disadvantaging women*”. When bringing her family, one participant felt that others took her “*less seriously as a scholar*”. Additionally, one mother felt frustrated about the decreasing quality of her work due to time pressures that also impacted her private life, which echoes experts encountering women who tried to prove their work capabilities regardless. Nevertheless, managing the workload while breastfeeding made women “*proud*”. Three found it easier to overcome challenges with “*stamina*”, intentions and a shift in priorities. Some noticed that excellent workplace support made them more comfortable as a mother and a more productive and satisfied employee.

### Policies and legal rights

#### Knowledge of policies and legal rights

Experts suggested that breastfeeding employees were not aware of their rights and that employers would not provide information. This was confirmed by mothers of which only one received direct guidance. Hence, women informed themselves online and via breastfeeding colleagues, friends or lactation specialists, but emphasised colleagues’ and superiors’ lack of awareness:

*“About the legal hours or so, they were not even aware. .. I’m quite sure that most employers, at least at the university, don’t know what your rights are, or they don’t find it important. .. you have to search for yourself and you don’t receive any information.”* (5A).

Half of the participants would have liked to be directly informed about entitlements and available facilities upon or prior to returning. Information packages on lactation rooms, sending photos or visiting spaces beforehand would have diminished uncertainties. Some women found it essential to firstly ask about personal needs as a basis for further information to prevent pressure and stigmatising those that could or did not wish to breastfeed. Four mothers underlined the role of Human Resources for receiving information and addressing experienced barriers, two suggested additional guidance from lactation consultants, and one woman recommended the occupational health physician to discuss general well-being. Having been informed beforehand, one participant commented that it impacted whether mothers could exercise their rights:

*“They* [universities] *should encourage to use it and not just give the information out when you ask for it, but they should be quite open and let everybody know that this is the situation and you can use this kind of time and you have this facility, so you can. .. make use of it.”* (13A).

#### Exercising rights

Unable to use appropriate lactation facilities and 25% paid working time, most employees emphasised that laws were not put into practice, resulting in the feeling that breastfeeding support was no priority. One employee called the gap between what was promised by law and on university websites, and what was practically provided, a *“mess”*. Another participant criticised the lack of implementation despite universities’ awareness of breastfeeding’s benefits:

*“They* [universities] *all know the numbers but, yeah, it’s so strange that they don’t practice what they preach.”* (10A).

However, women tried finding their own solutions rather than defending their rights. Mothers did not want to be regarded as “*less useful*” by employers and did neither have the energy nor the time to “*start this fight*”. Instead, laws needed to be enforced, but there was no monitoring and compliance mechanism:

*“We have all these laws, but there is no one checking. And it’s all up to the individual woman. .. if you have these laws, you have to comply to them. And you have to have measures to- to check.”* (1E).

Therefore, one mother suggested that the university’s ombudsman could make employers aware of issues addressed by employees, while experts suggested a phone number for those experiencing difficulties or a fine for employers.

Despite generally approving the laws, many respondents emphasised the importance of needs-based approaches, allowing mothers to find individual solutions. Additionally, three criticised the nine months cut-off point of the 25% policy as mothers should decide themselves about continuing. Reflecting on existing government campaigns, experts did not notice their utilisation and questioned the effectiveness. Instead, they advocated for more political commitment to normalise breastfeeding through awareness-raising, more media attention, enforcing the International Code of Marketing of Breast-Milk Substitutes, supporting WHO recommendations, and improving parental leave.

#### Maternity leave

Participants were unanimous that maternity leave was too short for breastfeeding and experts perceived this as the primary barrier to continued breastfeeding, particularly for bottle refusers. While one mother felt “*ready*” to return to work, others extended their maternity leave. Half advocated for a gradual shift and many started working fewer hours. Comparing it to employees returning after a significant health event, the leave was found insufficient for postpartum recovery:

*“It’s very sudden going back to work. .. for some, it* [birth] *was really a big health, major event. Physical-wise, maybe mental health-wise, maybe both, that you have to recover from quite quickly in the time that you have to breastfeed, which is taking lots of energy, in which you don’t sleep much. .. , it’s asking a lot.”* (8A).

Further, some felt uneasy about leaving their infant at day-care after three months. Hence, Dutch maternity leave was described as “*terrible*” and “*random*”, making all participants call for an extension. While two proposed six months, one expert noted that nine months pregnancy required nine months recovery. Another expert added that the one week paid paternal leave needed to be increased since partners provided crucial support.

## Discussion

This research identified that multidimensional factors influenced breastfeeding after returning to employment. Participants had more negative than positive experiences and encountered barriers, which could adversely affect maternal and child health. Participants who were not breastfeeding during data collection provided work-related reasons for ceasing breastfeeding or pumping earlier than recommended. Some reported problems with their milk production, which hampered direct breastfeeding. Thus, it is hypothesised that returning to employment can impede breastfeeding continuation. Interrelated themes reflected the Social Ecological Model’s dimensions, highlighting its applicability for workplace breastfeeding.

Firstly, the organisational dimension concerning inflexible working hours and unadjusted workloads was dominant, resulting in perceived pressures to function as before. These competing demands following “ecological transitions” [25] to work affected mothers’ mental health. Similarly, academic employees in Australia determined inflexible and demanding workloads as central aspects to stop breastfeeding [34]. Furthermore, many participants experienced lacking, inappropriate and inaccessible facilities inhibiting a comfortable and hygienic pumping. Previous Dutch research proposed that good lactation room quality could positively influence employees’ decision to pump, highlighting its importance [35].

Secondly, at the interpersonal dimension, those in male-dominated environments perceived less encouragement and were more hesitant to communicate about obstacles than mothers with more female colleagues, reflecting the social constructivist paradigm that social contexts influenced perceptions [24]. In line with previous research finding that Spanish university employees with female superiors were more likely to breastfeed [36], present participants in female-dominated environments were more positive and highlighted the importance of sharing experiences. Hence, social workplace support might buffer the impact of employment on breastfeeding. However, people might not see the importance of supporting breastfeeding employees, especially when lacking knowledge regarding its benefits for infants, mothers and employers through higher job satisfaction and less absenteeism. This is also associated with discourses around academic mothers, contradictory expectations and social perceptions at community level where breastfeeding is not normalised. Van Amsterdam [37] explained these tensions through a Foucauldian perspective, arguing that academic employees were disciplined to accommodate implicit “masculine norms” of control, competitive power relations and organisational boundaries, which breastfeeding disturbed. This was reflected by some participants who attempted to be a productive employee by separating breastfeeding from work as much as possible, feeling uncomfortable and almost apologetic when breastfeeding disrupted their work. Concerning childcare at the community level, one mother with access to on-campus day-care emphasised the benefits thereof, similar to Australian university employees [38]. Hence, this possibility could facilitate breastfeeding.

At the intrapersonal level, those feeling strongly about continuing breastfeeding found it easier to overcome barriers through their own solutions. Previous research identified self-efficacy and intentions as predictors of breastfeeding duration [39], and attitude towards pumping as a predictor of university employees’ intention to pump [40]. This indicates that breastfeeding knowledge is an important parameter, which limits the comparability of present participants’ breastfeeding duration to other Dutch study participants.

Finally, regarding the public policy dimension, most were not informed by universities about the facilities or legal rights and were unable to utilise these, suggesting an implementation gap. Dutch maternity leave was found insufficient for postpartum recovery and not supportive for exclusive breastfeeding, demonstrated by some participants’ postponed returns and their longer breastfeeding duration. Comparable to the present unanimous viewpoint, research at a Dutch maternity unit noted that all participants found maternity leave too short [41].

### Recommendations

Despite some settings being more enabling than others, universities need to increase institutional commitments to support breastfeeding employees. Easily accessible, multiple, high quality lactation rooms must be arranged. Universities could provide maps of lactation facilities for more transparency and mothers should receive their own room keys to not depend on others. Breastfeeding must become part of working cultures through practical support, open communication, making explicit whom women can turn to, and showing interest in maternal well-being. Human Resources should provide tailored information upfront, which is crucial to support parents before “ecological transitions” [25]. Furthermore, mothers should be able to organise their work flexibly and gradually, go to the child to feed, bring the child to work or work from home as required. Schedules, workloads and outputs must be adjusted through a needs-based approach in line with Directive (EU) 2019/1158 [42] on work-life balance for gender equality at work through parents’ and carers’ right to flexible work organisation. Thus, universities should close the implementation gap, fulfil legal obligations and put maternal rights into practice. This requires monitoring, for instance by Human Resources and the university’s ombudsman at the institutional level, while also auditing at the national level. Finally, more political commitment for normalising breastfeeding, enforcing regulations and supportive parental leave is necessary. Previous research indicates that maternity leave length is positively associated with breastfeeding duration [43, 44]. The International Labour Organization [45] called for paid maternity leave of at least 18 weeks, whereas the WHO and United Nations Children’s Fund [46] advocated for six months.

Although impact assessments for the proposal of the work-life balance Directive determined legislative provisions for breastfeeding breaks and facilities beneficial for mothers, employers and governments regarding labour markets and healthcare systems, there is no specific EU policy to date [47, 48]. Based on article 153 (1) of the Treaty on the Functioning of the European Union [49] there is, however, potential for EU added value through policy guidance. In the academic environment, increasing partnerships of tertiary education institutions, such as the European Universities [50], highlight the importance of common guidelines to protect breastfeeding employees. Since workplace breastfeeding or pumping is already not functioning well at university level, the situation is presumably worse for women in lower socio-economic jobs, underlining the need to put the issue higher on agendas.

### Strengths and limitations

Appearing to be the first qualitative research targeting academic employees in the Netherlands, this study analysed the complexity of workplace breastfeeding and highlighted the need for multidimensional interventions. The participation of women from five universities provided a comprehensive view and demonstrated that mothers faced similar issues, regardless of individual characteristics and academic positions. Despite data saturation, the applicability of findings to other academic employees should be considered with caution since universities were not equally represented. Further, the reliance on self-reporting information might have introduced recall bias, two interviews were time-limited, and a few struggled with English. Interview quality was impacted as most participated from home and simultaneously supervised their child(ren), although women felt presumably comfortable to talk openly in their homes.

Although the focus on academic employees limits generalisability, this study presents a basis for research targeting other socio-economic groups and investigating the association between breastfeeding duration, work-related factors and parental leave. Future studies should pay attention to socio-economic groups that might be less aware and flexible than highly educated mothers, making workplace support and information-provision essential. Additionally, further research is needed for observing cultural differences. Notably, the sharp decline in Dutch exclusive breastfeeding rates shortly after childbirth has to be investigated for early interventions.

## Conclusions

Despite legal entitlements, mothers employed at Dutch universities struggle to breastfeed or pump at work, which could harm maternal and child health. To enable infant feeding in line with WHO recommendations and to ease the transition back into employment, universities need to institutionalise breastfeeding support at physical, interpersonal and organisational level, thereby promoting family-friendly work environments and gender equality. In addition to workplaces as critical points for interventions, increasing breastfeeding rates necessitates national promotion regarding awareness-raising, enforcing regulations, monitoring, and parental leave. Thus, workplace breastfeeding and pumping needs to be facilitated and normalised so that employment is not the reason why women cease breastfeeding or breastfeed in secret.

## Data Availability

Qualitative interview data is not made available due to privacy concern.

## Abbreviations

WHO: World Health Organization

## References

[1] World Health Organization, Regional Office for Europe. WHO European Region has lowest global breastfeeding rates [Internet]. Denmark: WHO Regional Office for Europe; [updated 5 Aug 2015]. https://www.euro.who.int/en/health-topics/Life-stages/maternal-and-newborn-health/news/news/2015/08/who-european-region-has-lowest-global-breastfeeding-rates. Accessed 25 Aug 2020.

[2] Peeters D, Lanting C, van Wouwe K (2015). Peiling melkvoeding van zuigelingen 2015 [Infant milk feeding survey 2015].

[3] Buijssen M, Jajou R, van Kessel FGB, Vonk Noordegraaf-Schouten MJM, Wijga AH, van Rossum CTM. Health effects of breastfeeding: an update (RIVM Report 2015–0043). Bilthoven: National Institute for Public Health and Environment (RIVM); 2015.

[4] Horta BL, Victora CG (2013). Short-term effects of breastfeeding: a systematic review on the benefits of breastfeeding on diarrhoea and pneumonia mortality.

[5] Horta BL, Loret de Mola C, Victora CG (2015). Long-term consequences of breastfeeding on cholesterol, obesity, systolic blood pressure and type 2 diabetes: a systematic review and meta-analysis. Acta Paediatr.

[6] Victora CG, Bahl R, Barros AJD, França GVA, Horton S, Krasevec J, Murch S, Sankar MJ, Walker N, Rollins NC (2016). Breastfeeding in the 21st century: epidemiology, mechanisms, and lifelong effect. Lancet..

[7] Walters DD, Phan LTH, Mathisen R (2019). The cost of not breastfeeding: global results from a new tool. Health Policy Plan.

[8] Rollins NC, Bhandari N, Hajeebhoy N, Horton S, Lutter CK, Martines JC, Piwoz EG, Richter LM, Victora CG (2016). Why invest, and what it will take to improve breastfeeding practices?. Lancet..

[9] World Health Organization, United Nations Children’s Fund. Global nutrition targets 2025: breastfeeding policy brief (WHO/NMH/NHD/14.7). Geneva: World Health Organization; 2014. https://www.who.int/nutrition/publications/globaltargets2025_policybrief_breastfeeding/en/. Accessed 22 Sep 2020.

[10] Baby Friendly Nederland [Baby Friendly Netherlands]. Baby Friendly stopt certificering [Baby Friendly stops certification] [Internet]. Grootebroek: Baby Friendly Nederland [updated 21 Nov 2018]. https://www.babyfriendlynederland.nl/nieuws/baby-friendly-stopt-certificering. Accessed 22 Sep 2020.

[11] Theurich MA, Davanzo R, Busck-Rasmussen M, Díaz-Gómez NM, Brennan C, Kylberg E, Bærug A, McHugh L, Weikert C, Abraham K, Koletzko B (2019). Breastfeeding rates and programs in Europe: a survey of 11 national breastfeeding committees and representatives. J Pediatr Gastroenterol Nutr.

[12] World Health Organization (1981). International code of Marketing of Breast-milk Substitutes.

[13] World Health Organization (2018). Marketing of Breast-milk Substitutes: National Implementation of the international code (status report 2018).

[14] Voedingscentrum [The Netherlands Nutrition Centre]. Borstvoeding werkt! (persmap 2019) [Breastfeeding works! (press kit 2019)] [internet]. Den Haag: Voedingscentrum. https://www.voedingscentrum.nl/nl/pers/persmappen/borstvoeding-werkt-2019.aspx. .

[15] Arora A, Manohar N, Hector D, Bhole S, Hayen A, Eastwood J, Scott JA (2020). Determinants for early introduction of complementary foods in Australian infants: findings from the HSHK birth cohort study. Nutr J.

[16] Gallagher L, Begley C, Clarke M (2016). Determinants of breastfeeding initiation in Ireland. Irish J Med Sci.

[17] Tarrant RC, Younger KM, Sheridan-Pereira M, Kearney JM (2011). Factors associated with duration of breastfeeding in Ireland: potential areas for improvement. J Human Lact.

[18] Lanting C, van Wouwe J, Reijneveld S (2005). Infant milk feeding practices in the Netherlands and associated factors. Acta Paediatr.

[19] Ministry of the Interior and Kingdom Relations. The legal status of civil servants (AVT10/BZK99520). Den Haag: Central Government Personnel and Organisation Department, Ministry of the Interior and Kingdom Relations; 2010.

[20] Jurviste U, Prpic M, Sabbati G. At a glance infographic: maternity and paternity leave in the EU. European Parliamentary Research Service; 2019. https://www.europarl.europa.eu/RegData/etudes/ATAG/2019/635586/EPRS_ATA(2019)635586_EN.pdf. .

[21] Arbeidstijdenwet, §4.3. Vrouwelijke werknemers, 4:8 Voedingsrecht [Working Hours Act, §4.3. Female employees, 4:8 Feeding right], Act of November 23, 1995. https://wetten.overheid.nl/BWBR0007671/2020-01-01#Hoofdstuk4. Accessed 30 Sep 2020.

[22] Kools EJ, Thijs C, de Vries H (2005). The behavioral determinants of breast-feeding in the Netherlands: predictors for the initiation of breast-feeding. Health Educ Behav.

[23] Perez SA, van den Brakel M, Portegijs W. Welke gevolgen heeft ouderschap voor werk en economische zelfstandigheid? [What are the consequences of parenthood for work and economic independence?] In: Portegijs, W, van den Brakel M, editors. Emancipatiemonitor 2018 [Emancipation monitor 2018]. Den Haag: Centraal Bureau voor de Statistiek/Sociaal en Cultureel Planbureau [Statistics Netherlands/The Netherlands Institute for Social Research]; 2018. (p. 62–68).

[24] Polit DF, Beck CT (2017). Nursing research: generating and assessing evidence for nursing practice.

[25] Bronfenbrenner U (1979). The ecology of human development.

[26] McLeroy KR, Bibeau D, Steckler A, Glanz K (1988). An ecological perspective on health promotion programs. Health Educ Q.

[27] Vereniging van Universiteiten [Association of Universities in the Netherlands]. University staff [Internet]. Den Haag: Vereniging van Universiteiten [updated 13 Aug 2020]. https://www.vsnu.nl/en_GB/university-employees. Accessed 22 Sep 2020.

[28] Vereniging van Universiteiten [Association of Universities in the Netherlands]. Ratio of permanent to temporary staff [Internet]. Den Haag: Vereniging van Universiteiten [updated: 13 Aug 2020]. https://www.vsnu.nl/en_GB/f_c_verhouding_vast_tijdelijk.html. Accessed 22 Sep 2020.

[29] Vereniging van Universiteiten [Association of Universities in the Netherlands]. Female academic staff [Internet]. Den Haag: Vereniging van Universiteiten [updated 13 Aug 2020]. https://www.vsnu.nl/en_GB/f_c_ontwikkeling_aandeel_vrouwen.html. Accessed 22 Sep 2020.

[30] Hernández PT, Callahan S (2008). Attributions of breastfeeding determinants in a French population. Birth..

[31] Hoskins CN, Mariano C (2004). Research in nursing and health: understanding and using quantitative and qualitative methods.

[32] Galletta A, Cross WE (2013). Mastering the semi-structured interview and beyond: from research design to analysis and publication.

[33] Braun V, Clarke V (2006). Using thematic analysis in psychology. Qual Res Psychol.

[34] Gilmour C, Monk H, Hall H (2013). Breastfeeding mothers returning to work: experiences of women at one university in Victoria, Australia. Breastfeed Rev.

[35] Van Dellen S, Wisse B, Mobach M, Dijkstra A. The impact of lactation room quality in facilitating the combination of breastfeeding and work. 2018. Poster session presented at Heymans symposium. https://www.rug.nl/research/portal/publications/the-impact-of-lactation-room-quality-in-facilitating-the-combination-of-breastfeeding-and-work(be095130-66b4-4ef0-be85-9b8987a814e0).html. .

[36] Leon-Larios F, Pinero-Pinto E, Arnedillo-Sanchez S, Ruiz-Ferron C, Casado-Mejia R, Benitez-Lugo M (2019). Female employees’ perception of breastfeeding-friendly support in a public university in Spain. Public Health Nurs.

[37] Van Amsterdam N (2015). Othering the ‘leaky body’. An autoethnographic story about expressing breast milk in the workplace. Cult Organ.

[38] Smith J, Javanparast S, Craig L (2017). Bringing babies and breasts into workplaces: support for breastfeeding mothers in workplaces and childcare services at the Australian National University. Breastfeed Rev.

[39] Wallenborn JT, Perera RA, Wheeler DC, Lu J, Masho SW (2019). Workplace support and breastfeeding duration: the mediating effect of breastfeeding intention and self-efficacy. Birth..

[40] Bai YK, Dinour LM, Pope GA (2016). Determinants of the intention to pump breast milk on a university campus. J Midwifery Womens Health.

[41] Oosterhoff AT (2015). Women’s perceptions, knowledge and breastfeeding decision-making: linking theory to qualitative empirical data [dissertation].

[42] Directive (EU) 2019/1158 of the European Parliament and of the Council of 20 June 2019 on work-life balance for parents and carers and repealing Council Directive 2010/18/EU.

[43] Navarro-Rosenblatt D, Garmendia ML (2018). Maternity leave and its impact on breastfeeding: a review of the literature. Breastfeed Med.

[44] De Lauzon-Guillain B, Thierry X, Bois C, Bournez M, Davisse-Paturet C, Dufourg MN (2019). Maternity or parental leave and breastfeeding duration: results from the ELFE cohort. Matern Child Nutr.

[45] International Labour Organization. R191 - Maternity protection recommendation, 2000 (No. 191). 2000.

[46] World Health Organization, United Nations Children’s Fund. Advocacy brief: breastfeeding and family-friendly policies (WHO/NMH/NHD/19.23). 2019.

[47] European Commission (2017). Study on the costs and benefits of possible EU measures to facilitate work-life balance for parents and care givers: final report.

[48] European Commission (2017). Impact assessment accompanying the document proposal for a directive of the European Parliament and of the council on work-life balance for parents and carers and repealing council directive 2010/18/EU Brussels [SWD (2017) 202 final].

[49] Consolidated version of the treaty on the functioning of the European Union 2012/C 326/01 [26 Oct 2012]. Off J Eur Union. 2012;C326:1–390.

[50] European Commission. European Universities: a key pillar of the European education area 2019 [Internet]. https://ec.europa.eu/education/sites/education/files/document-library-docs/european-universities-initiative-factsheet.pdf. Accessed 22 Sep 2020.

